# Father Involvement in Infant Parenting in an Ethnically Diverse Community Sample: Predicting Paternal Depressive Symptoms

**DOI:** 10.3389/fpsyt.2020.578688

**Published:** 2020-09-23

**Authors:** Olajide N. Bamishigbin, Dawn K. Wilson, Demetrius A. Abshire, Cilia Mejia-Lancheros, Christine Dunkel Schetter

**Affiliations:** ^1^Department of Psychology, California State University, Long Beach, Long Beach, CA, United States; ^2^Department of Psychology, University of South Carolina, Columbus, SC, United States; ^3^College of Nursing, University of South Carolina, Columbus, SC, United States; ^4^MAP Centre for Urban Health Solutions, St. Michael's Hospital, Unity Health, Toronto, ON, Canada; ^5^Department of Psychology, University of California, Los Angeles, Westwood, CA, United States

**Keywords:** fathers, depressive symptoms, paternal involvement, parenting self-efficacy, low-income fathers, community-based participatory research

## Abstract

Early paternal involvement in infant care is beneficial to child and maternal health, and possibly for paternal mental health. The purpose of the present study was to examine the relationship between fathers’ involvement in early infant parenting and their depressive symptoms during the infant’s first year in a sample of 881 low-income Black, Hispanic, and White fathers recruited from five sites in the United States (urban, mixed urban/suburban, rural). Home interviews at 1 month after birth assessed three concepts based on prior research and community input: (1) time spent with the infant, (2) parenting self-efficacy, (3) material support for the baby. Paternal depressive symptoms at 1, 6, and 12 months after the birth of a child were assessed with the Edinburgh Postpartum Depression Scale. Generalized estimating equations tested whether the three indicators of father involvement at 1 month after birth predicted lower subsequent paternal depressive symptoms controlling for social and demographic variables. For fathers, greater time spent with the infant, parenting self-efficacy, and material support were all significantly associated with lower paternal depressive symptoms during the first year. When risk of depression (scores > 9) was examined, only parenting self-efficacy among fathers was associated with higher likelihood of clinical depression. Findings have implications for future research on mechanisms linking paternal involvement and paternal mental health, and for possible paid paternal leave policies in the future.

## Introduction

Paternal depressive symptoms following the birth of a child are now a topic of increasing interest (1, 2). Estimates from three meta-analyses indicate that approximately 8 to 10 percent of men experience depression during the transition period to parenting and during early fatherhood (3–5), with the highest prevalence at 3 to 6 months after birth (3, 4). The prevalence of prenatal and postnatal depression among fathers is approximately twice as high as the prevalence of depression among men in general (6). The substantial proportion of fathers experiencing postpartum depression has important implications not only for fathers, who deserve attention, but also for maternal and child health and well-being (7, 8). Fathers often exert a strong influence on family life and functioning (9), and paternal depressive symptoms have been associated with adverse child and maternal mental health outcomes such as depression and psychiatric disorders (4, 10–13). The present study incorporates fathers and their experiences into our understanding of the family unit during the time following a birth.

One factor that is implicated in the phenomenon of paternal depression is low levels of paternal involvement with children (14). The meaning and characterization of paternal involvement has evolved over the past few decades with early perspectives emphasizing the father’s role as a moral teacher and provider (15–17) to more contemporary perspectives emphasizing the importance of actual time fathers are involved in the different domains of accessibility, engagement, and responsibility (16, 18). Accessibility reflects the fathers’ presence and availability to the child irrespective of the type or quality of actual interactions. Engagement reflects the ways in which fathers directly interact with the child including time spent in activities such as caregiving and play. Responsibility reflects involvement with supervisory parenting activities such as scheduling medical appointments and choosing a daycare. The present study focuses specifically on more contemporary paternal involvement constructs that incorporate paternal engagement and responsibility for the infant.

Previous research has demonstrated that greater paternal involvement is associated with positive family outcomes. Specifically, greater paternal involvement has been associated with lower maternal depressive symptoms (19, 20) and higher maternal life satisfaction (19). During infancy, greater paternal involvement has also been associated with lower rates of infant mortality (21), more secure father-child attachment (22), lower likelihood of infant cognitive delay (23) and higher child IQ at three years of age (24). In addition, investigators have shown that paternal depression, maternal depression, and the amount of time fathers spend interacting with their children during infancy reduces child behavioral problems in kindergarten (25). The conceptualization of fatherhood used in the current study may be limited to fathers in Western cultures. What constitutes acceptable levels of paternal involvement may also differ depending on cultural background (26, 27). Even given these cultural differences, previous research clearly demonstrates that paternal involvement may play an important role in adjustment for the mother and the child.

To our knowledge, no study has examined the link between early paternal involvement and later paternal depressive symptoms following the birth of a child. Previous studies have examined the associations between father involvement and depressive symptoms using cross-sectional study designs. One study of preteen sons of nonresidential Black fathers found that lower paternal involvement was cross-sectionally associated with higher levels of paternal depressive symptoms in controlled analyses [e.g., (28)]. Furthermore, a recent study reported that fathers who were depressed were more withdrawn when playing with their infants in comparison to non-depressed fathers (29), and were more likely to neglect their children (30). Another study reported that paternal depressive symptoms were not associated with paternal involvement such as caregiving and play time (31). Thus, it is not known whether father involvement in early infancy has protective benefits with respect to later paternal depressive symptoms, during the year following a child’s birth.

Based on previous conceptualizations of father involvement, this study focused on three aspects of involvement – the amount of time fathers spent with the infant, parenting self-efficacy, and the father’s provision of material support for the baby– in predicting paternal depression over the first year after the birth of a child.

### Time Spent With New Infant

Fathers who are unable to spend enough time with the infants may be at increased risk of feelings of sadness or dissatisfaction over the early years of their children’s lives—especially in the first year when infants grow and change so much (32). Time spent with their children may be particularly relevant for low-income fathers, as these fathers emphasize the importance of “being there” for their children even when direct contact is infrequent due to social, economic, and other barriers (33). Many fathers also view the provider role as providing material support such as money and supplies, in addition to spending time with their children (33). Although the amount of time that fathers spend with their children does not reflect the nature and quality of father-child interactions (16), the amount of time fathers spend with their infants is a more objective indicator of father accessibility and a precondition for types of father involvement. Several previous investigators have assessed time that fathers were engaged with their infants. However, few have examined the associations of time spent with infants and paternal (or any family member’s) health-related outcomes (17, 34).

### Parenting Self-Efficacy

Parenting self-efficacy, or self-confidence, which reflects a father’s ability to care for offspring, is an important determinant of paternal involvement (17, 35). To our knowledge, no study has evaluated whether parenting self-efficacy is related to depressive symptoms over time in low-income fathers from racially and ethnically diverse backgrounds. One longitudinal study of 86 fathers from the first trimester to 6 months after the birth of a child found that greater parenting self-efficacy was correlated with lower depressive symptoms (36). Cross-sectionally, parenting self-efficacy has been positively associated with father involvement (37, 38). Self-efficacy is important because it may increase a father’s level of involvement, which in turn may lead to stable parenting, and better paternal mental health outcomes. Thus, the present study incorporated a self-efficacy measure of father involvement to further our understanding of paternal well-being in this longitudinal study.

### Material Support

Consistent with a historical perspective on fathers (17), scholars have argued that the procurement of economic resources and being a provider for the family are also important (39), and especially when fathers and mothers are not in an ongoing marital or close relationship. Others have noted the importance of financial support as being a form of paternal involvement within the responsibility domain (18) and have suggested that provision of material support by fathers is critical aspect of father involvement (40).

The present study extends our understanding of how early father involvement relates to paternal depressive symptoms longitudinally within a large, racially and ethnically diverse sample of low-income fathers. While it is expected that parenting self-efficacy, time spent with infant, and provision of material support are intercorrelated to some extent, these concepts reflect distinct aspects of paternal involvement. Therefore, in this study, we evaluated whether time spent with a new infant, parenting self-efficacy, and material support at 1 month after the birth of a child are associated with paternal depressive symptoms over the first 12 months of parenting among fathers, an understudied population. Specifically, it was hypothesized that 1 month after the birth of a child, more time spent with infants, greater parenting self-efficacy, and more material support provided for the child would predict fewer depressive symptoms in fathers over the first year after a child’s birth.

## Methods

### Community Child Health Network Procedure

The Community Child Health Network (CCHN) is a multi-site network of interdisciplinary researchers funded by the Eunice Kennedy Shriver National Institute of Child and Health Development to investigate biopsychosocial mechanisms underlying racial/ethnic disparities in maternal and child health (41). Researchers used community-based participatory research methods, which involves collaborations with members of the community through every phrase of research from planning to dissemination in order to examine risk and resilience in low-income mothers and fathers in five sites across the United States (Los Angeles, California; Washington, D.C.; Baltimore, Maryland; Lake County, Illinois; several rural counties in North Carolina). The first three sites (Los Angeles, Washington D.C., and Baltimore) were urban environments, Lake County was a suburban environment, and the counties in North Carolina were rural sites. Researchers received IRB approval at each study site.

Mothers were recruited in the hospital after the birth of their child in four sites (Los Angeles, Lake County, Baltimore, and Washington D.C.) and in prenatal clinics in one site (North Carolina). Mothers were eligible if they were African American, Latina, or non-Hispanic White. Fathers were interviewed only if the mothers gave permission to the researchers to contact the fathers. Then, fathers were approached and invited to participate in the study. All procedures for mothers and fathers were approved by the various hospital and university institutions, and informed consent procedures were followed to enroll fathers and mothers into the study. Recruiters and interviewers were trained staff and research assistants from the partnered academic and community institutions.

Mothers and fathers were interviewed separately in their homes at 1 month (T1), 6 months (T2), and 12 months after the birth of a child (T3) [For further details on CCHN study, see (41, 42)]. The current study focuses on the subset of fathers who completed interviews at 1 month (T1) and 12 months (T3) after their child’s birth.

### Participants

At 1 month (T1), 2,510 mothers agreed to participate. Of these, 1,923 gave permission to contact fathers and of those, 1,758 fathers agreed to participate. This group was comprised of fathers from diverse racial/ethnic backgrounds, however, due to small subgroup sizes, multi-racial fathers (n = 38), Asian-American and Pacific Islander fathers (n = 14), and fathers for whom race/ethnicity was missing and could not be determined (n = 37) were dropped from analyses leaving a sample size of 1,669. Of these, 53 percent (n = 881) completed the T3 depressive symptoms measure. Thus, the final sample size is 881 fathers. Descriptive characteristics including age, racial/ethnic background, marital and cohabitation status, levels of education, employment status, and site are reported in **Table 1**.

**Table 1:** Demographic characteristics of fathers (observed data).

Main characteristics	N = 881	% or mean (± SD) and median (IQR)	N (%) of missing values
**Father’s demographic factors at 1 month after offspring born**			
**Age** (years)	**793**	29.54 (7.13)	88 (9.99)
		28.22 (23.70–33.76)	
**Racial/ethnic background**	**881**		
African-American or Black	393	44.61	
Latino or Hispanic	242	27.47	
White or Caucasian	246	27.92	
**Place of birth**	**790**		
US-born	599	75.82	91 (10.33)
Foreign-born	191	24.18	
**Family factors at 1 month after offspring born**			
**Marital/cohabiting status**	**812**		
Married and cohabiting	359	44.21	69 (7.83)
Not married but cohabiting	276	33.99	
Not married not cohabiting	163	20.07	
Married but not cohabiting	14	1.72	
**Cohabiting with the newborn ** ** offspring**	**781**		
No	119	15.24	100 (11.35)
Yes	662	84.76	
**Having other offspring**	**783**		
No	606	77.39	98 (11.12)
Yes	177	22.61	
**Socioeconomic characteristics 1 month after offspring born**			
**Education level**	**779**		
Less than high school	183	23.49	102 (11.58)
HS, GED, certificate	318	40.82	
Some college	124	15.92	
4-year degree or higher	154	19.77	
**Employment level**	**715**		
Employed full or part time	516	72.17	166 (18.84)
Unemployed	137	19.16	
Other (military, student)	62	8.67	
**Rurality level of the recruitment ** ** area of the father’s offspring ** ** mother**	**881**		
Urban/Suburuban area^a^	732	83.09	
Rural area^a^	149	16.91	

^a^Urban/Suburban: offspring’s mothers recruited in Baltimore, Chicago, Los Angeles, and Washington DC). Rural area: offspring’s mothers recruited in North Carolina.

Bolded numbers reflect the number of cases for which we had full data.

Analyses were conducted to determine whether there were any significant differences between the 881 Black, Latino, and White fathers in the current study who completed the T3 depressive symptoms measure and the 788 Black, Latino, and White fathers who did not. Independent samples t-tests revealed no significant differences between these groups of fathers on employment status, whether the father had prior children, parenting self-efficacy, time spent with infant, material support for the baby, or depressive symptoms at T1 or T2. However, fathers who completed the T3 depressive symptoms measure were significantly older (*t* (1,181) = 3.68, *p* <.001) and had more years of education (*t* (1,313) = 3.24, *p* <.01), compared to fathers who did not complete the T3 measure of depressive symptoms. Chi-square analyses found that fathers who were White (*X*^2^ (2, *N* = 1,669) = 16.79, *p* <.001), from Chicago (*X*^2^ (4, *N* = 1,659) = 36.03, *p* <.001), married to their baby’s mother (*X*^2^ (1, *N* = 1,467) = 26.89, *p* <.001), and cohabiting with their baby’s mother (*X*^2^ (1, = 1,413) = 16.21, *p* <.001) were significantly more likely to complete the T3 depressive symptoms measure than Black fathers, fathers from Los Angeles, unmarried fathers, and non-cohabiting fathers, respectively.

### Measures

#### Demographic Data

Several socio-demographic characteristics were considered in this study as covariates including race/ethnicity, paternal age, place of birth (foreign-born vs. U.S. born), marital and cohabitation status with the baby’s mother, cohabitation status with the baby, level of education, employment status, and type of recruitment site (rural vs. not rural).

#### Father Involvement Measures

The measures for fathers were designed by a CCHN subcommittee of researchers and community partners including experts on father research. The work was informed by the Fragile Families and Child Well-Being Study, a longitudinal birth cohort study that followed nearly 4,900 families in large U.S. cities between 1998 and 2000 (43). All father measures used in this study were adapted from instruments in the Fragile Families and Child Well-Being study and provided to CCHN by members of the FFCWS team. Following review by a CCHN community-partnered measurement subcommittee and piloting a subset of items were used in this study.

##### Time Spent With Infant

Fathers responded to four items about the time they spent with their infants: 1) On an average weekday from Monday to Friday, do you spend any waking hours with [BABY]? 2) On an average weekday from Monday to Friday, do you spend time alone with [BABY]? 3) On an average weekend day meaning Saturday and Sunday, do you spend any waking hours with [BABY]? 4) On an average weekend day meaning Saturday and Sunday, do you spend time alone with [BABY]? Responses to each question were 0 (*no*) or 1 (*yes*) and were summed. Scores range from 0 to 4 and a higher score indicates more time spent with the infant.

##### Parenting Self-Efficacy

Paternal self-efficacy in parenting tasks was measured with six items. This scale was adapted from the FFCWS. In the present study, fathers were asked “how confident or comfortable you feel when you” 1) hold baby, 2) put baby to sleep, 3) wash or bathe baby, 4) change baby’s diaper, 5) feed baby, and 6) soothe baby when he/she is upset. Responses range from 1 (*not at all*) to 4 (*very much*). Responses were averaged with higher scores indicative of greater parenting self-efficacy. Cronbach’s alpha coefficient for these items for the full sample of fathers at 1 month was.68.

##### Material Support

A total of 10 items were used to measure the degree of material support fathers provided for their baby. Fathers reported how often they provided a) baby clothing, b) medicine for baby, c) baby furniture or equipment, d) childcare items, such as diapers, baby wipes, e) food, f) babysitting, g) money, h) health insurance, i) toys, and j) other. Possible responses were 0 (*no*), 1 (*yes, occasionally*), and 2 (*yes, regularly*). Scores were summed and range from 0 to 20. Higher scores reflect greater material support. Cronbach’s alpha was.77.

### Depressive Symptoms Measures

The primary outcome was the fathers’ depressive symptoms scores, which were measured with the 10-item Edinburgh Postpartum Depression Scale administered at 1, 6, and 12 months after the birth of the child. This scale was originally designed for use with mothers but has been validated in fathers (44) as well as racial/ethnic minorities (45). Responses range from 0 (*no, not at all*) to 3 (*yes, quite a lot*). The response values were summed into an overall score, which in our study population ranged from 0 to 20. Higher scores indicate greater depressive symptoms. This scale had good reliability at T1 (Cronbach’s alpha = .78) and T3 (Cronbach’s alpha = 80).

Depressive symptoms were also examined in exploratory analyses using a cutoff for likely depressive illness. Researchers have used different scale cut-offs to indicate possible or probable diagnosis of depression including over 8 (46), over 9 (44, 47), and over 10 (48). In the current study, the most conventional cut-off score of ≥ 9 was used.

### Data Analysis Plan

All analyses were performed at a 95% confidence interval using Stata Software (version 16). First, descriptive statistics were computed for demographic and socioeconomic characteristics as well as the fathers’ parenting involvement measures and depression scores. Second, we conducted preliminary analyses of the associations between the main three father involvement measures and depression values over the one-year follow-up period using the Generalized estimating equation [GEE; (49)] which accounts for repeated measures. Some study variables had incomplete or missing data (see **Supplementary Table 1**), ranging from 8% (marital and cohabitation status) to depressive symptoms at T2 (25%). Hence, we imputed missing values using Multiple Imputation (MI) *via* chained equations (50). We compared the distribution and proportion of the observed, imputed, and completed values (51) which showed good appropriateness of the imputed values (see examples of the comparison in **Supplementary Figures 1–3**, and **Supplementary Tables 2–8**). Third, we repeated the analysis with the imputed data to test the associations between each of the father’s parenting involvement measures (material support, parenting self-efficacy, and time spent with infant) with the continuous depression scale scores and the dichotomous depression cut-off (≥ 9). These data are presented and discussed in the present paper. We fitted the GEE models with the Gaussian family, Identity link, and exchangeable correlation with robust covariance when the EPDS scale scores were analyzed as outcomes. We fitted the GEE models with Binomial family, Logit link, and exchangeable correlation with robust covariance when the nine-cut-off values of the EPDS scale were analyzed as an outcome. We adjusted the crude association (Model 1) for each fathers parenting measures with the depression outcomes (EPDS score and nine-cut-off of EPDS scale values) in the following core adjusted models: Model 2, adjusted for age, and racial/ethnic background; Model 3, added the marital/cohabiting status, and having other offspring; Model 4, introduced educational level; Model 5, added rurality level of the recruitment area of the father’s offspring mother. Due to multi-collinearity, in Adjusted Model 6, we replaced the marital/cohabiting status variable of Model 5 with cohabiting status with the new-born offspring. In Model 7, we substituted the educational level variable of Model 5 with employment status. Finally, in Model 8, we replaced the race/ethnicity variable of Model 5 with place of birth. We performed these variable substitutions as these variables are too highly correlated to include in the same model. Further, each of the substituted variables may have different confounding effects on the assessed association.

Finally, we tested potential interaction effects between three parenting involvement measures using three-way interaction terms (Material support*Self-efficacy*Time spent with Infant). Since the interaction terms were not statistically significant (*p* > 0.05), we present the final models without interaction factors.

## Results

### Descriptive Statistics of Main Study Variables

**Table 2** displays the descriptive statistics for the parental involvement measures and depression symptoms scores variables. On average, fathers had relatively high score values on parenting self-efficacy (*M* = 3.61, *SD* = .46), with scores reflecting that fathers feel “pretty much” to “very much” confident or comfortable in executing new-born offspring’s tasks. For the provision of material support to the newborn, the mean was 14.29 (SD = 3.97), showing that fathers provided support for the infant between occasionally and regularly. The majority of fathers (63%) reported spending waking hours with their children on weekdays and weekends and alone time with their children on weekdays and weekends. The average father’s scores on the depressive symptoms’ scales over the first year of parenting ranged from 3.71 at 1 month to 4.07 at 12 months after the child was born. Based on the cut-off of 9, the percentage of fathers with scores suggestive of clinical depression was 10%, 15%, and 12% at T1, T2, and T3, respectively.

**Table 2:** Descriptive Characteristics of fathers’ parenting measures scores and depression symptoms scores (observed data).

Main characteristics	N = 881	% or mean (± SD) and median (IQR)	n (%) of missing values
**Father’s parenting involvement measures at 1 month after offspring born**.			
** Father’s parenting self-efficacy ** ** (mean score, range: 1**–**4)**	**779**	3.61 (0.46)	102 (11.58)
		3.80 (3.40–4.0)	
** Father’s provision of material ** ** support to the offspring (total ** ** score, ** ** range: 0 to 20)**	**782**	14.29 (3.97)	99 (11.24)
		15.00 (12.00–18.00)	
** Father’s average time spent with ** ** the new-born offspring during the ** ** week^a^**	**782**		
≤ 2 days	145	18.54	99 (11.24)
3 days	144	18.41	
4 or more days	493	63.04	
**Father’s depression values over the first years of parenting**			
** Father’s EPDS scale^b^ at 1 month ** ** after the offspring born (total score, ** ** range: 0**–**30)**	**716**	3.71 (3.83)	165 (18.73)
		3.00 (1.00–6.00)	
Depression not likely (EPDS scale < 9 cutoff)	642	89.66	
Depression possible (EPDS scale **≥** 9 cutoff)	74	10.34	
** Father’s EPDS scale^b^ at 6 months ** ** after the offspring born (total ** ** range: 0**–**30)**	**662**	4.49 (4.05)	219 (24.86%)
		4.00 (1.00–6.00)	
Depression not likely (EPDS scale < 9 cutoff)	564	85.20	
Depression possible (EPDS scale **≥** 9 cutoff)	98	14.80	
** Father’s EPDS scale^b^ at 12 months ** ** after the offspring born (total ** ** score, range: 0**–**30)**	**881**	4.07 (4.02)	
		3.00 (1.00–6.00)	
Depression not likely (EPDS scale < 9 cutoff)	771	87.51	
Depression possible (EPDS scale **≥** 9 cutoff)	110	12.49	

^a^Includes weekdays and weekend days.

^b^Edinburgh Postnatal Depression Scale.

Bolded numbers reflect the number of cases for which we had full data.

### Correlations Between Father Involvement Variables

Pearson’s correlation coefficients showed significant but weak associations among the father involvement variables. Greater parenting self-efficacy was positively correlated with greater provision of tangible support (*r* = .18) and more time spent with the infant (*r* = .18). In addition, greater tangible support was also associated with more time spent with infant (*r* = .18).

### Fathers’ Parenting Self-Efficacy and Depressive Symptoms Scores

The unadjusted and adjusted associations of fathers’ parenting self-efficacy scores at 1 month after the offspring born with their depression scores (EPDS scale, range 0–20) over the first year of parenting are presented in **Table 3**. A one-point increase in the fathers’ self-efficacy score was significantly associated with lower values of the EPDS scale, even when adjusted for age, racial/ethnic background, marital/cohabiting status, having other offspring, educational level, and rurality level of the recruitment area of the father’s offspring mother (**Table 3**, Model 5=Adjusted coefficient and 95% CI: −1.652, −2.203 to −1.101). Similar results were observed when accounting for cohabiting with the new-born offspring (**Table 3**, Model 6), employment status (**Table 3**, Model 7), and place of birth (**Table 3**, Model 8).

**Table 3:** Unadjusted and adjusted associations between father’s parenting self-efficacy with depression scores (EPDS scale) over the first year of parenting (completed imputed data).

N = 881 (Observations: 2,643)	Father’ depression scores (EPDS scale, range 0–20) over the first year of parenting		
	Coefficient	95%CI	p-value
**^a^Model 1**			
Father’s parenting self-efficacy (mean score, range: 1–4)	−1.641	−2.175 to −1.108	<0.001
**^b^Model 2**			
Father’s parenting self-efficacy (mean score, range: 1–4)	−1.609	−2.157 to −1.062	<0.001
**^c^Model 3**			
Father’s parenting self-efficacy (mean score, range: 1–4)	−1.631	−2.175 to −1.087	<0.001
**^d^Model 4**			
Father’s parenting self-efficacy (mean score, range: 1–4)	−1.613	−2.157 to −1.069	<0.001
**^e^Model 5**			
Father’s parenting self-efficacy (mean score, range: 1–4)	−1.652	−2.203 to −1.101	<0.001
**^f^Model 6**			
Father’s parenting self-efficacy (mean score, range: 1–4)	−1.611	−2.164 to −1.058	<0.001
**^g^Model 7**			
Father’s parenting self-efficacy (mean score, range: 1–4)	−1.708	−2.251 to −1.166	<0.001
**^h^Model 8**			
Father’s parenting self-efficacy (mean score, range: 1–4)	−1.749	−2.306 to −1.193	<0.001

^a^Unadjusted association. Wald Prob. chi2: <0.001.

^b^Adjusted for father’s age and racial/ethnic background. Wald Prob. chi2: <0.001.

^c^Adjusted for father’s age, racial/ethnic background, marital/cohabiting status, and having other offspring. Wald Prob. chi2: <0.001.

^d^Adjusted for father’s age, racial/ethnic background, marital/cohabiting status, having other offspring, and educational level. Wald Prob. chi2: <0.001.

^e^Adjusted for father’s age, racial/ethnic background, marital/cohabiting status, having other offspring, educational level, and rurality level of the recruitment area of the father’s offspring mother. Wald Prob. chi2: <0.001.

^f^Model 5, substituting marital/cohabiting status by new-born offspring. Wald Prob. chi2: <0.001.

^g^Model 5, substituting educational status by employment status. Wald Prob. chi2: <0.001.

^h^Model 5, substituting racial/ethnic background by place of birth. Wald Prob. chi2: <0.001.

### Fathers’ Time Spent With the Newborn During the Week and Depressive Symptoms Scores

**Table 4** shows the unadjusted and adjusted association between time spent with the new-born during the week at 1 month after the offspring born and depression scores over the first years of parenting. After adjusting for age, racial/ethnic background, marital/cohabiting status, having other offspring, educational level, and rurality level of the recruitment area of the offspring mother, spending 4-days or more with the new-born child was significantly associated with lower depression scores (**Table 4**, model 5=Adjusted coefficient and 95% CI: −0.647, −1.274 to −0.021). Although this association was smaller in effect size when accounting for cohabiting status with the baby (**Table 4**, model 6), it remained significant when controlling for employment (**Table 4**, model 7), and place of birth (**Table 4**, model 8).

**Table 4:** Unadjusted and adjusted associations between the father’s average time spent with the new-born offspring during the week with depression scores (EPDS scale) over the first year of parenting (completed imputed data).

N=881 (Observations: 2, 643)	Father’s depression scores (EPDS scale, range 0–20) over the first year of parenting		
	Coefficient	95% CI	p-value
**^a^Model 1**			
Father’s average time spent with the new-born offspring during the week			
3 days (vs ≤ 2days)	−0.918	−1.722 to −0.114	0.025
4 days or more (vs ≤ 2days)	−0.920	−1.561 to −0.279	0.005
**^b^Model 2**			
Father’s average time spent with the new-born offspring during the week			
3 days (vs ≤ 2days)	−0.879	−1.674 to −0.084	0.030
4 days or more (vs ≤ 2days)	−0.787	−1.429 to −0.145	0.016
**^c^Model 3**			
Father’s average time spent with the new-born offspring during the week			
3 days (vs ≤ 2days)	−0.760	−1.531 to 0.012	0.054
4 days or more (vs ≤ 2days)	−0.666	−1.289 to −0.044	0.036
**^d^Model 4**			
Father’s average time spent with the new-born offspring during the week			
3 days (vs ≤ 2days)	−0.751	−1.528 to 0.026	0.058
4 days or more (vs ≤ 2days)	−0.637	−1.264 to −0.011	0.046
**^e^Model 5**			
Father’s average time spent with the new-born offspring during the week			
3 days (vs ≤ 2days)	−0.761	−1.538 to 0.015	0.055
4 days or more (vs ≤ 2days)	−0.647	−1.274 to −0.021	0.043
**^f^Model 6**			
Father’s average time spent with the new-born offspring during the week			
3 days (vs ≤ 2 days)	−0.728	−1.515 to 0.059	0.070
4 days or more (vs ≤ 2 days)	−0.602	−1.229 to 0.025	0.060
**^g^Model 7**			
Father’s average time spent with the new-born offspring during the week			
3 days (vs ≤ 2 days)	−0.760	−1.522 to 0.002	0.051
4 days or more (vs ≤ 2 days)	−0.776	−1.400 to −0.152	0.015
**^h^Model 8**			
Father’s average time spent with the new-born offspring during the week			
3 days (vs ≤ 2 days)	−0.781	−1.562 to −0.001	0.050
4 days or more (vs ≤ 2 days)	−0.733	−1.365 to −0.101	0.023

^a^Unadjusted association. Wald Prob. chi2: 0.018.

^b^Adjusted for father’s age and racial/ethnic background. Wald Prob. chi2: <0.001.

^c^Adjusted for father’s age, racial/ethnic background, marital/cohabiting status, and having other offspring. Wald Prob. chi2: 0.003.

^d^Adjusted for father’s age, racial/ethnic background, marital/cohabiting status, having other offspring, and educational level. Wald Prob. chi2: <0.001.

^e^Adjusted for father’s age, racial/ethnic background, marital/cohabiting status, having other offspring, educational level, and rurality level of the recruitment area of the father’s offspring mother. Wald Prob. chi2: <0.001.

^f^Model 5, substituting marital/cohabiting status by new-born offspring. Wald Prob. chi2: <0.001.

^g^Model 5, substituting educational status by employment status. Wald Prob. chi2: <0.001.

^h^Model 5, substituting racial/ethnic background by place of birth. Wald Prob. chi2: <0.001.

### Fathers’ Provision of Material Support to the Newborn and Depressive Symptoms Scores

**Table 5** displays the unadjusted and adjusted associations of fathers’ provision of material support score at 1 month after the offspring born with their depression scores (EPDS scale, range 0–20) over the first year of parenting. Greater material support was associated with lower depressive symptoms after accounting for age and racial/ethnic background (**Table 5**, model 2, coefficient and 95% CI: −0.109 to −0.172 to −0.046), marital/cohabiting status and having other children (**Table 5**, model 3, coefficient and 95% CI: −0.086, −0.146 to −0.025), educational level (**Table 5**, model 4, coefficient and 95% CI: −0.080, −0.142 to −0.019), and rurality level of the recruitment area (**Table 5**, model 5, coefficient and 95% CI: −0.081, −0.143 to −0.020). Similar estimations were observed when accounting for cohabiting with the new-born offspring (**Table 5**, model 6), employment status (**Table 5**, model 7), and place of birth (**Table 5**, model 8).

**Table 5:** Unadjusted and adjusted associations between father’s provision of material support to the offspring with depression scores (EPDS scale) over the first year of parenting (completed imputed data).

N=881 (Observations: 2, 643)	Father’ depression scores (EPDS scale, range 0–20) over the first year of parenting		
	Coefficient	95%CI	p-value
**^a^Model 1**			
Father’s provision of material support to the new-born offspring (total score, range: 0−20)	−0.122	−0.184 to −0.061	<0.001
**^b^Model 2**			
Father’s provision of material support to the new-born offspring (Total score, range: 0–20)	−0.109	−0.172 to −0.046	0.001
**^c^Model 3**			
Father’s provision of material support to the new-born offspring (Total score, range: 0–20)	−0.086	−0.146 to −0.025	0.006
**^d^Model 4**			
Father’s provision of material support to the new-born offspring (Total score, range: 0–20)	−0.080	−0.142 to −0.019	0.011
**^e^Model 5**			
Father’s provision of material support to the new-born offspring (Total score, range: 0–20)	−0.081	−0.143 to −0.020	0.010
**^f^Model 6**			
Father’s provision of material support to the new-born offspring (Total score, range: 0–20)	−0.081	−0.143 to −0.019	0.011
**^g^Model 7**			
Father’s provision of material support to the new-born offspring (Total score, range: 0–20)	−0.078	−0.140 to −0.017	0.013
**^h^Model 8**			
Father’s provision of material support to the new-born offspring (Total score, range: 0–20)	−0.085	−0.147 to −0.023	0.007

^a^Unadjusted association. Wald Prob. chi2: <0.001.

^b^Adjusted for father’s age and racial/ethnic background. Wald Prob. chi2: <0.001.

^c^Adjusted for father’s age, racial/ethnic background, marital/cohabiting status, and having other offspring. Wald Prob. chi2: <0.001.

^d^Adjusted for father’s age, racial/ethnic background, marital/cohabiting status, having other offspring, and educational level. Wald Prob. chi2: <0.001.

^e^Adjusted for father’s age, racial/ethnic background, marital/cohabiting status, having other offspring, educational level, and rurality level of the recruitment area of the father’s offspring mother. Wald Prob. chi2: <0.001.

^f^Model 5, substituting marital/cohabiting status by new-born offspring. Wald Prob. chi2: <0.001.

^g^Model 5, substituting educational status by employment status. Wald Prob. chi2: <0.001.

^h^Model 5, substituting racial/ethnic background by place of birth. Wald Prob. chi2: <0.001.

### Father Involvement Measures and the Likelihood for Depression

**Figure 1:**
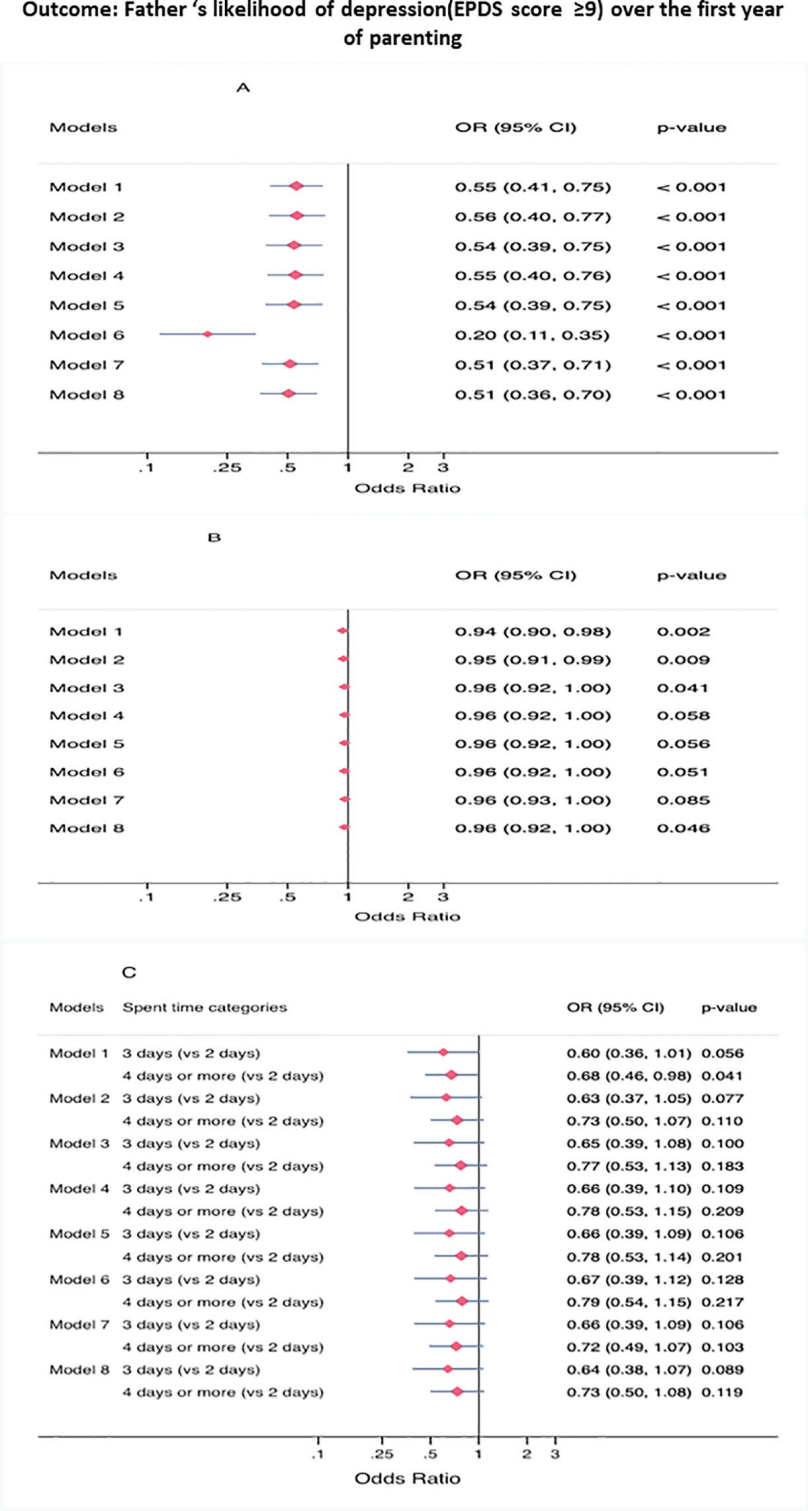
**(A)** Exposure: parenting self-efficacy, **(B)** Exposure: provision of material support, **(C)** Exposure: father's weekly time spent with the new-born. Unadjusted and adjusted associations of Father’s parenting self-efficacy, fathers’ provision of material support to the new-born, father’s average time spent with the new-born during the week with the EPDS scale ≥ 9 cut-off over the first year of parenting (completed imputed data, N=881, observations: 2,643). Model 1: Unadjusted association. Model 2: Adjusted for father's age and racial/ethnic background. Model 3: Adjusted for father's age, racial/ethnic background, marital/cohabiting status, and having other offspring. Model 4: Adjusted for father's age, racial/ethnic background, marital/cohabiting status, having other offspring, and educational level. Model 5: Adjusted for father's age, racial/ethnic background, marital/cohabiting status, having other offspring, educational level, and rurality level of the recruitment area of the father's offspring mother. Model 6: Model 5, substituting marital/cohabiting status by new-born offspring. Model 7: Model 5, substituting educational status by employment status. Model 8: Model 5, substituting racial/ethnic background by place of birth.

**Figure 1** displays the unadjusted and adjusted association of father parenting self-efficacy scores in executing new-born offspring’s tasks (**Figure 1A**), provision of material support to the new-born child (**Figure 1B**) and spent four or more days per week with their new-born child (**Figure 1C**) with the likelihood of depression using the apriori cutpoint (EPDS score: ≥ 9) over the first years of parenting. After controlling for all demographic, family, and socioeconomic factors, as well as rurality status of the recruitment area of the offspring mother, only higher values of father parenting self-efficacy scores were negatively associated with the likelihood of depression over the first year of parenting (**Figure 1A**). Father’s provision of material support to the new-born offspring and time spent with the new-born were not statistically significant after controlling for the covariates (**Figures 1B, C**).

## Discussion

This study examined the correspondence between early paternal involvement with a newborn infant at one month after birth and paternal depressive symptoms nearly one year later within a community sample of low-income and ethnically diverse fathers from five areas of the U.S. We found that fathers who spent more time with their infants, had greater parenting self-efficacy, and provided more material support for the baby one month after the birth reported significantly lower depressive symptoms when the child was one year of age. To the best of our knowledge, this study is the first to show longitudinal associations between greater perceived parenting self-efficacy and lower depressive symptoms, and specifically in low SES fathers from diverse racial/ethnic backgrounds. These findings suggest that paternal involvement is an important predictor of father’s mental health during the transition to fatherhood. Thus, parental involvement is an important for these men who were present at one month after birth, even though significant portions of them did not live with and were not married to the baby’s mother.

Parenting self-efficacy may be related to depressive symptoms as a result of higher parenting satisfaction. Previous research has shown that greater parenting self-efficacy is associated with greater parenting satisfaction in fathers (52), and has been associated with lower prevalence of paternal depressive symptoms (53). Fathers who feel competent as parents may therefore be more satisfied in their roles, and as a result, have fewer depressive symptoms. Research in fathers has also shown that when fathers rate their infants as less “fussy,” they report fewer depressive symptoms (54, 55), although other researchers have not replicated these findings (56). Additional research is needed to identify the underlying mechanisms through which greater parenting self-efficacy is associated with lower paternal depressive symptoms. Nonetheless, these findings suggest that it may be beneficial to foster the development of fathers’ parenting skills during prenatal and postnatal visits, as suggested by Salonen and colleagues (57). This may be particularly important for first time fathers who may have low parenting self-efficacy. Different online intervention programs have been successful in increasing parenting self-efficacy in fathers (58, 59). Adapting these programs and making them culturally sensitive and accessible to fathers from diverse racial/ethnic and socioeconomic backgrounds may be useful in reducing paternal depressive symptoms.

Regarding the link between parenting self-efficacy and parental depression, Jones and Prinz (60) note that “it is not altogether clear whether parenting self-efficacy functions consistently as an antecedent or contributor to parental depression, as a consequence of parental depression, or in a transactional relationship with parental depression. On the one hand, low parenting self-efficacy can contribute to maternal vulnerability for depression. Alternatively, depression can lead to lower maternal parenting self-efficacy” (p. 352). Although this was written with regard to mothers, it applies equally to fathers. Future research in this area should better understand the complex nature of their association.

Spending more time with a child one month after his or her birth was associated in this study with fewer paternal depressive symptoms nearly one year later. A potential explanation for this relationship may be related to employment. Fathers who work more hours and make more money spend less time with their child (61) and working more hours has been associated with greater paternal depressive symptoms (62, 63). Another study found that fathers who were better able to cope with major stressors in their life, such as work, spent more time with their kids (64). However, we accounted for employment status in our analysis and the observed associations between time spent with the newborn infant and lower father’s depression symptoms at one year remained significant.

Fathers who provided more material support in the form of diapers, toys, clothing, and food at one month after the birth of their baby had lower depressive symptoms at one year later. These findings held even after controlling for socioeconomic status and family factors such cohabitation with the child and with the child’s mother. Considerable research has demonstrated that the provider role is an important identity for father’s perceptions of themselves as well as mother’s perceptions of fathers (33, 39, 65–67). As a result, fathers who are less able to provide support, typically measured in the form of money, may have poor self-image, resulting in depressed mood. There is some evidence to support this as previous research indicated that fathers who were less able to provide economic support reported more depressive symptoms (68, 69). Previous research also demonstrates that when fathers provide greater financial and instrumental support, their children engage in fewer behavior problems (70, 71), which, in turn, may be associated with lower paternal depressive symptoms. Another reason why material support may be associated with fewer depressive symptoms is that material support is a type of social support well known to be beneficial not only to recipients but also to those providing it (72–74). A longitudinal study with over 700 families from diverse racial/ethnic backgrounds demonstrated bi-directional effects between paternal depressive symptoms and child behavior problems [e.g., internalizing behaviors, externalizing behaviors; (75)]. Thus, while paternal depressive symptoms affect children’s well-being, children’s well-being also affects paternal depressive symptoms.

There are a number of important implications for public health policies given the findings reported in this study. For example, greater time spent with the infant was associated with lower depressive symptoms, which has implication for designing policies to support opportunity to spend more time with their children without penalizing fathers who work. One avenue for increased time spent with kids may be paid paternal leave. Paid paternal leave for fathers of young children may be beneficial for the entire family unit (76). Research has shown that fathers in countries with more paternal paid leave spend significantly more time with their children (77) and fathers who take longer leave when their children are born spend more time on child-related tasks 9 months later (78). In light of the results of the current study, paid leave may also play a role in decreasing levels of paternal depressive symptoms, although more research is needed to elucidate this link and associated mechanisms.

There may several pathways linking early paternal involvement to later depressive symptoms in fathers. Specifically, lower depressive symptoms have been associated with changes in hormonal and neural functioning (79) and increased oxytocin levels (80). Oxytocin, known as the “love hormone” due to its links with social bonding and reproduction, increases in fathers following the birth of the child and after father-child interactions (81). Although we did not assess actual father-child interactions in our study, fathers who spend more time with their infants may have been engaging in positive interactions that affect hormonal and neural functioning, which may protect against future depressive symptoms. Furthermore, while the current study did not assess father-infant attachment, it may play a significant role in paternal mental health. Previous studies have demonstrated that stronger infant-parent attachment is associated with many positive outcomes for the child (82) and fathers feel valued when they have stronger attachments with their young children (83). In addition, father-infant attachment is associated with greater father involvement (84). Future research is needed to better elucidate these potential mechanisms.

The current study is not without limitations. Although we assessed different domains of involvement, we did not directly assess engagement with the child or quality of engagement in any way. Several research studies have shown that the quality and nature of time spent with the child is important for child well-being among non-resident fathers (85, 86). In addition, this study did not control for factors related to the child’s mother (e.g., maternal depression, serious health problems) that may have contributed to father’s depression, which was beyond the scope of this study and can be examined in future. A large body of research has shown that paternal depressive symptoms are associated with greater maternal depression (10) and poorer relationship quality (87, 88), and thus future research is warranted on this topic. The current study design also does not permit causal inferences although longitudinal data with temporal precedence were used. Although we used a repeated measure analysis approach to account for associations between depressive symptoms within subjects at different timepoints, we did not control for fathers’ depressive symptoms during pregnancy or history of depression as this information was not collected. Thus, there is the possibility that some fathers were already depressed before their child born, and a reverse causality may be present such that their depression led to lower involvement at 1 month. Finally, the fathers in this study only participated if the researchers received consent from the mother. Although the findings may not be generalizable to fathers in other socioeconomic and cultural settings, this study is among the first studies to involve a relatively large community sample of low-income fathers from diverse racial/ethnic backgrounds.

In conclusion, this study demonstrates that greater paternal involvement may benefit paternal mental health in low income ethnically and racially diverse fathers. Specifically, greater involvement in the form of time spent with the newborn, parenting self-efficacy, and ability to provide material support were all implicated as aspects of fathering that predicted lower depressive symptoms in fathers, and self-efficacy predicted risk of depressive disease. More research is needed to address these important constructs of father involvement to better understand how to improve paternal mental health and overall wellbeing. This study suggests that these factors associated with depression in fatherhood may be addressed by increasing skills in parenting, improving or enhancing ways for fathers to spend time with their children, as well as enabling fathers to provide material support for their children. Future researchers should consider designing and testing interventions to assess the impact of paid paternal leave and increases in parenting self-efficacy skills on paternal depression in ethnically diverse populations.

## Data Availability Statement

Publicly available datasets were analyzed in this study. These data can be found here: https://dash.nichd.nih.gov/study/1649.

## Ethics Statement

The studies involving human participants were reviewed and approved by Institutional Review Boards for Johns Hopkins University, University of California, Los Angeles, Cedars-Sinai Medical Center, University of North Carolina, Northshore University Health System, Evanston Northwestern Healthcare Research Institute, Georgetown University. The patients/participants provided their written informed consent to participate in this study.

## Author Contributions

OB, DW, DA, CD, and CM-L all contributed to the writing of this manuscript. CD and CCHN collected the original data. DW and DA wrote the *Introduction*. OB wrote the *Methods* section. CM-L wrote the *Results* sections. OB and CD wrote the *Discussion* section. CM-L conducted the statistical analysis of the paper.

## Funding

This article is a product of the Child Community Health Research Network (CCHN). The CCHN was supported through cooperative agreements with the Eunice Kennedy Shriver National Institute of Child Health and Human Development (U HD44207, U HD44219, U HD44226, U HD44245, U HD44253, U HD54791, U HD54019, U HD44226-05S1, U HD44245-06S1, R03 HD59584) and the National Institute for Nursing Research (U NR008929). For more information on CCHN network sites, see https://www.nichd.nih.gov/research/supported/Pages/cchn.aspx. DA was supported by the National Institute on Minority Health and Health Disparities of the National Institutes of Health under award number K23MD013899.

## Conflict of Interest

The authors declare that the research was conducted in the absence of any commercial or financial relationships that could be construed as a potential conflict of interest.
