# Supplementary material for: Father Involvement in Infant Parenting in an Ethnically Diverse Community Sample: Predicting Paternal Depressive Symptoms
Source: Front Psychiatry. 2020 Sep 23;11:578688. doi: 10.3389/fpsyt.2020.578688 (PMC7538507; doi:10.3389/fpsyt.2020.578688)
Supplement: Supplementary file 1 [file DataSheet_1.pdf]

Supplementary Table 1. Complete, Incomplete and Imputed values for the participant of the study.

Variable imputed; 25 imputation dataset	Complete (n)	Incomplete (n)	Imputed (n)	Total (N)
Age	793	88	88	881
EPDS T1	716	165	165	881
EPDS T2	662	219	219	881
Confidence score	779	102	102	881
Tangible support score	782	99	99	881
Weekly days spent with the child	812	69	69	881
Place of birth	790	91	91	881
Marital/cohabiting status	812	69	69	881
Cohabiting with the child	781	100	100	881
Other children	783	98	98	881
Education level	779	102	102	881
Employment status	715	166	166	881

Supplementary Table 2. Comparison of the proportions of the observed, imputed and completed values for the father's weekly days spent with the child at T1 in the last 5 imputed datasets.

		Proportions		
Imputed dataset	Weekly days spent with the child	Observed	Imputed	Complete
m=21	<=2 days	0.185	0.212	0.188
	3 days	0.184	0.141	0.179
	4 days or more	0.630	0.646	0.632
m=22	<=2 days	0.185	0.273	0.195
	3 days	0.184	0.111	0.176
	4 days or more	0.630	0.616	0.629
m=23	<=2 days	0.185	0.242	0.192
	3 days	0.184	0.202	0.186
	4 days or more	0.630	0.556	0.622
m=24	<=2 days	0.185	0.141	0.180
	3 days	0.184	0.202	0.186
	4 days or more	0.630	0.657	0.633
m=25	<=2 days	0.185	0.141	0.180
	3 days	0.184	0.202	0.186
	4 days or more	0.184	0.131	0.178

Supplementary Table 3. Comparison of the proportions of the observed, imputed and completed values for the father's place of born in the last 5 imputed datasets.

		Proportions		
**Imputed dataset**	**Place of birth**	**Observed**	**Imputed**	**Complete**
m=21	US born	0.758	0.791	0.762
	Foreign born	0.242	0.209	0.238
m=22	US born	0.758	0.780	0.760
	Foreign born	0.242	0.220	0.240
m=23	US born	0.758	0.714	0.754
	Foreign born	0.242	0.286	0.246
m=24	US born	0.758	0.747	0.757
	Foreign born	0.242	0.253	0.243
m=25	US born	0.758	0.758	0.758
	Foreign born	0.242	0.242	0.242

Supplementary Table 4. Comparison of the proportions of the observed, imputed and completed values for the father's marital/cohabiting status at T1 in the last 5 imputed datasets.

		Proportions		
**Imputed dataset**	**Marital/cohabiting status**	**Observed**	**Imputed**	**Complete**
m=21	Married and cohabiting	0.442	0.420	0.440
	Not married but cohabiting	0.340	0.275	0.335
	Not married not cohabiting	0.201	0.261	0.205
	Married but not cohabiting	0.017	0.043	0.019
m=22	Married and cohabiting	0.442	0.377	0.437
	Not married but cohabiting	0.340	0.420	0.346
	Not married not cohabiting	0.201	0.174	0.199
	Married but not cohabiting	0.017	0.029	0.018
m=23	Married and cohabiting	0.442	0.304	0.431
	Not married but cohabiting	0.340	0.464	0.350
	Not married not cohabiting	0.201	0.217	0.202
	Married but not cohabiting	0.017	0.014	0.017
m=24	Married and cohabiting	0.442	0.391	0.438
	Not married but cohabiting	0.340	0.348	0.341
	Not married not cohabiting	0.201	0.232	0.203
	Married but not cohabiting	0.017	0.029	0.018
m=25	Married and cohabiting	0.442	0.290	0.430
	Not married but cohabiting	0.340	0.478	0.351
	Not married not cohabiting	0.201	0.159	0.198
	Married but not cohabiting	0.017	0.072	0.02

Supplementary Table 5. Comparison of the proportions of the observed, imputed and completed values for the father's cohabiting with the child status at T1 in the last 5 imputed datasets.

		Proportions		
**Imputed dataset**	**Cohabiting with the child status**	**Observed**	**Imputed**	**Complete**
m=21	No	0.152	0.270	0.166
	Yes	0.848	0.730	0.834
m=22	No	0.152	0.260	0.165
	Yes	0.848	0.740	0.835
m=23	No	0.152	0.220	0.160
	Yes	0.848	0.780	0.840
m=24	No	0.152	0.200	0.158
	Yes	0.848	0.800	0.842
m=25	No	0.152	0.310	0.170
	Yes	0.848	0.690	0.830

Supplementary Table 6. Comparison of the proportions of the observed, imputed and completed values for the father's having other children at T1 in the last 5 imputed datasets.

		Proportions		
**Imputed dataset**	**Having other children**	**Observed**	**Imputed**	**Complete**
m=21	No	0.774	0.724	0.768
	Yes	0.226	0.276	0.232
m=22	No	0.774	0.755	0.772
	Yes	0.226	0.245	0.228
m=23	No	0.774	0.796	0.776
	Yes	0.226	0.204	0.224
m=24	No	0.774	0.745	0.771
	Yes	0.226	0.255	0.229
m=25	No	0.774	0.704	0.766
	Yes	0.226	0.296	0.234

Supplementary Table 7. Comparison of the proportions of the observed, imputed and completed values for the father's educational level at T1 in the last 5 imputed datasets.

		Proportions		
**Imputed dataset**	**Educational level**	**Observed**	**Imputed**	**Complete**
m=21	Less than High School	0.235	0.255	0.237
	HS, GED, Certificate	0.408	0.392	0.406
	Some College	0.159	0.167	0.160
	4-year degree or higher	0.198	0.186	0.196
m=22	Less than High School	0.442	0.377	0.437
	HS, GED, Certificate	0.408	0.529	0.422
	Some College	0.159	0.157	0.159
	4-year degree or higher	0.198	0.127	0.190
m=23	Less than High School	0.235	0.314	0.244
	HS, GED, Certificate	0.408	0.343	0.401
	Some College	0.159	0.147	0.158
	4-year degree or higher	0.198	0.196	0.198
m=24	Less than High School	0.235	0.235	0.235
	HS, GED, Certificate	0.408	0.382	0.405
	Some College	0.159	0.186	0.162
	4-year degree or higher	0.198	0.196	0.198
m=25	Less than High School	0.235	0.196	0.230
	HS, GED, Certificate	0.408	0.333	0.400
	Some College	0.159	0.255	0.170
	4-year degree or higher	0.198	0.216	0.200

Supplementary Table 8. Comparison of the proportions of the observed, imputed and completed values for the father's employment status at T1 in the last 5 imputed datasets.

		Proportions		
**Imputed dataset**	**Weekly days spent with the child**	**Observed**	**Imputed**	**Complete**
m=21	Employed full or part-time	0.722	0.590	0.697
	Unemployed	0.192	0.277	0.208
	Other (military, student)	0.087	0.133	0.095
m=22	Employed full or part-time	0.722	0.651	0.708
	Unemployed	0.192	0.211	0.195
	Other (military, student)	0.087	0.139	0.096
m=23	Employed full or part-time	0.722	0.620	0.703
	Unemployed	0.192	0.283	0.209
	Other (military, student)	0.087	0.096	0.089
m=24	Employed full or part-time	0.722	0.620	0.703
	Unemployed	0.192	0.253	0.203
	Other (military, student)	0.087	0.127	0.094
m=25	Employed full or part-time	0.722	0.675	0.713
	Unemployed	0.192	0.241	0.201
	Other (military, student)	0.087	0.084	0.086
