# Supplementary material for: Father Involvement in Infant Parenting in an Ethnically Diverse Community Sample: Predicting Paternal Depressive Symptoms
Source: Front Psychiatry. 2020 Sep 23;11:578688. doi: 10.3389/fpsyt.2020.578688 (PMC7538507; doi:10.3389/fpsyt.2020.578688)
Supplement: Supplementary file 2 [file DataSheet_2.pdf]

Supplementary Figure 1. Comparison of the distributions of the observed, imputed and completed values for the father's EPDS score at T1 and T2 in the last 5 imputed datasets.

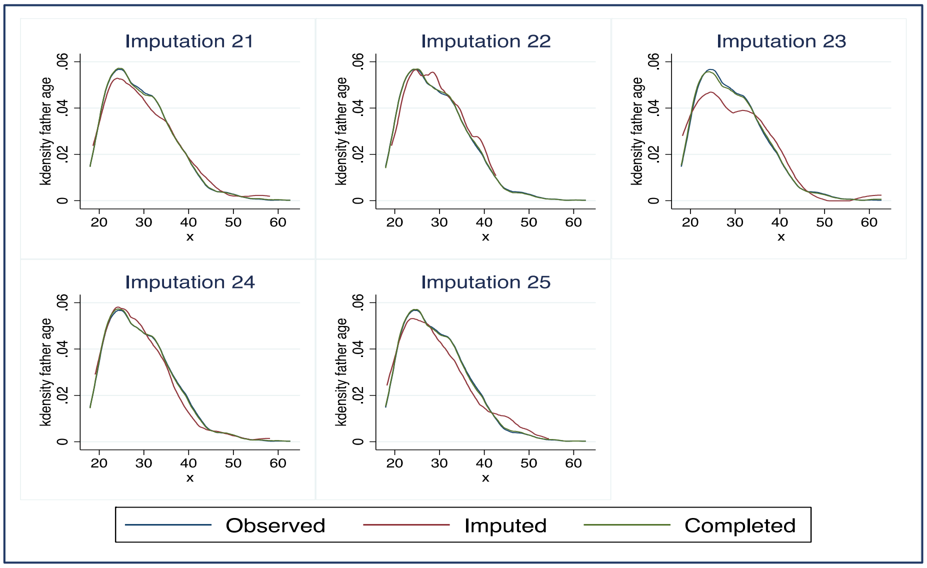

Age	
Imputed dataset	Kolmogorov-Smirnov test p-value (Combined K-S)
m=21	0.992
m=22	0.843
m=23	0.311
m=24	0.792
m=25	0.739

Supplementary Figure 2. Comparison of the distributions of the observed, imputed and completed values for the father's EPDS score at T1 and T2 in the last 5 imputed datasets.

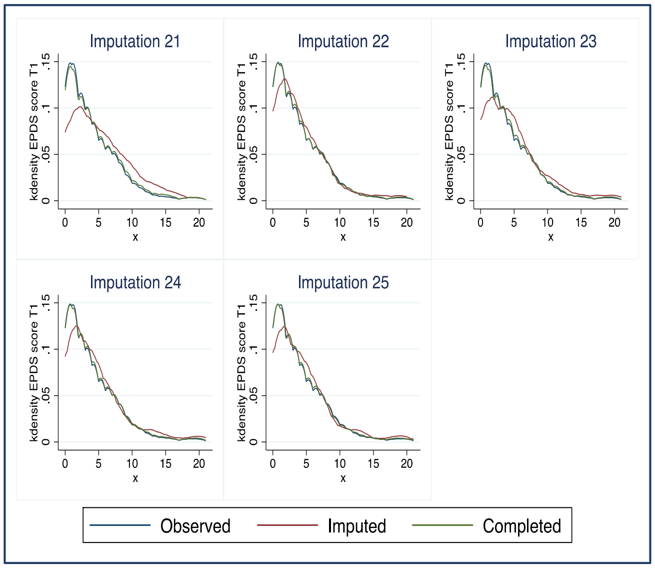

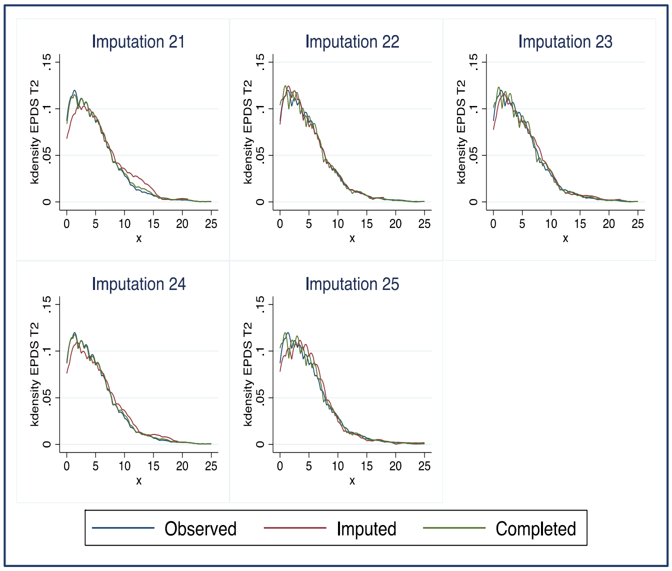

EPDS score at T1	
Imputed dataset	Kolmogorov-Smirnov test p-value (Combined K-S)
m=21	0.056
m=22	0.737
m=23	0.080
m=24	0.375
m=25	0.722

EPDS score at T2	
Imputed dataset	Kolmogorov-Smirnov test p-value (Combined K-S)
m=21	0.150
m=22	1.000
m=23	0.896
m=24	0.615
m=25	0.501

Supplementary Figure 3. Comparison of the distributions of the observed, imputed and completed values for the father's confidence score and tangible support score at T1 in the last 5 imputed datasets.

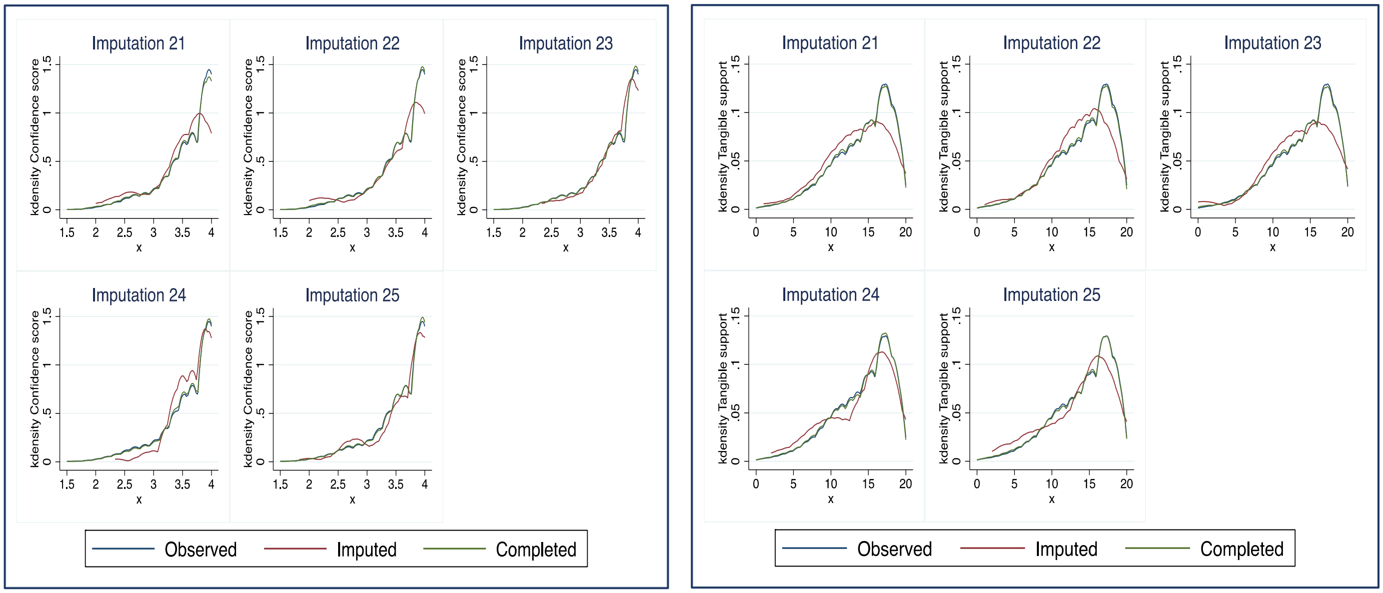

Confidence score	
Imputed dataset	Kolmogorov-Smirnov test p-value (Combined K-S)
m=21	0.091
m=22	0.996
m=23	0.940
m=24	0.170
m=25	0.922

Tangible support score	
Imputed dataset	Kolmogorov-Smirnov test p-value (Combined K-S)
m=21	0.246
m=22	0.141
m=23	0.346
m=24	0.747
m=25	0.470
